# Effects of Maternal Low-Energy Diet during Gestation on Intestinal Morphology, Disaccharidase Activity, and Immune Response to Lipopolysaccharide Challenge in Pig Offspring

**DOI:** 10.3390/nu9101115

**Published:** 2017-10-13

**Authors:** Yuling Chen, Daolin Mou, Liang Hu, Jie Zhen, Lianqiang Che, Zhengfeng Fang, Shengyu Xu, Yan Lin, Bin Feng, Jian Li, De Wu

**Affiliations:** Institute of Animal Nutrition, Sichuan Agricultural University, No. 211, Huimin Road, Wenjiang District, Chengdu 611130, China; chenyuling1994@foxmail.com (Y.C.); mdl19920101@foxmail.com (D.M.); 18728158035@139.com (L.H.); CY199404@foxmail.com (J.Z.); clianqiang@hotmail.com (L.C.); fangzhengfeng@hotmail.com (Z.F.); shengyu_x@hotmail.com (S.X.); able588@163.com (Y.L.); fengb123d@163.com (B.F.); lijian522@hotmail.com (J.L.)

**Keywords:** low energy diet, intestinal, immunity, maternal, offspring

## Abstract

Maternal nutrition during gestation is involved in the offspring’s intestinal development and immunity. The aim of this study was to (1) determine the effects of maternal energy on intestinal digestion and absorption function in offspring, using pigs as a model; and (2) to evaluate the potential effect and mechanisms of maternal energy in modulating immune responses of lipopolysaccharide (LPS)-challenged piglets. After mating, thirty-six nine-parity sows (Landrace × Yorkshire), body weight (BW) (initial body weight 233.56 ± 2.77 kg) were allocated to two dietary treatment groups; a control diet (CON) group and a low-energy diet (LED) group. The nutrient levels of the CON were based on the nutrient recommendations by the National Research Council (NRC, 2012), and contained 3.40 MCal digestible energy (DE)/kg diet and 7.3% crude protein; while the LED contained 3.00 MCal DE/kg diet. The dietary treatments were introduced from day 1 of gestation to farrowing. Intestine samples were collected from the pigs’ offspring at birth, and at weaning (day 28 post-birth). At weaning, male pigs from control and LED groups were intraperitoneally injected with LPS (50 μg/kg body weight) or saline (*n* = 6), and sacrificed at 4 h post-injection to collect blood, intestine and digesta samples for biochemical analysis. The results indicated that the maternal LED markedly decreased the BW, small intestinal weight, and the ratio of jejunum and ileum villus height to crypt depth in the offspring. Moreover, the activities of lactase and sucrase in newborn piglets’ intestine, and sucrase and maltase in weaning piglet intestine were markedly decreased by the maternal LED. In addition, maternal LED significantly increased the mRNA relative expression of ileal *IL-6* and *TNF-α* in newborn piglets. Plasma IL-1β concentration and colonic *Escherichia coli* amount were affected by maternal diet (*p* < 0.05) and LPS challenge (*p* < 0.001). Maternal LED significant increased the mRNA relative expression of ileal *TLR-4*, *IL-1β* and *NF-κB* as well as decreased *ZO-1* in weaning pigs after LPS challenge (*p* < 0.05). In conclusion, decreasing energy intake could suppress the offspring’s intestinal digestion and absorption function, and increase the susceptibility of weaning piglets to LPS challenge.

## 1. Introduction

Maternal malnutrition in pregnancy can cause maternal endocrine and metabolic disorders, further damaging the growth of the placenta, thereby affecting the mother-to-fetus nutrient transport and weakening the growth and development of the fetus [1]. Pregnancy malnutrition will not only delay the growth of the fetus, but also damage many of the fetus’ organs, as well as their organizational structure and function [2]. In animal and human nutrition, the gastrointestinal tract is responsible for the introduction of nutrients into the body cells. However, the intestine is not only a major part of the digestion and absorption of nutrients, but is also a key part of the process of immune challenge, because it is constantly interacting with foreign substances [3]. A large amount of experimental data has shown that nutritional factors, including energy, protein, fat and fatty acids, affect intestinal function [4], and some studies have also shown that maternal malnutrition during pregnancy may result in poor development of the intestinal tract and intestinal function damage [5,6]. Due to development and growth constraints, compared with normal newborn piglets, intrauterine growth restriction (IUGR) piglets often manifest immune dysfunctions [7,8]. Neonates with IUGR show substantially higher rates of perinatal mortality and morbidity, partly due to impairment of cell proliferation, and nutrient digestion, absorption, and metabolism in the small intestine (SI) [9,10,11]. At birth, the intestinal and immune systems of the newborn piglets are complete, but the functions of the small intestine and the immune system are immature, and are susceptible to infection and damage by harmful antigens. Interactions between the microbiota and the host are being considered as potential players in the early programming of gut functions. Increasing evidence indicates that the gut microbiota programs the functions and metabolism of host organs, including the GIT [12,13]. Thus, early nutrition plays a very important role in protecting the development of the small intestine and adjusting the immune response after the challenge of the antigen [14]. However, the effect of maternal low-energy intake on intestinal immune function of offspring is not known. Many studies on early programming have been conducted in rodents and sheep, and a recent literature survey suggests that swine are a good translational model for humans [15]. In this study, therefore, we investigated whether providing maternal low-energy intake would affect intestinal development and immune function in offspring.

## 2. Materials and Methods

The study protocol was reviewed and approved by the Care and Use Committee of Sichuan Agricultural University, and followed the current laws of animal protection (Ethic Approval Code: SCAUAC201408-3).

### 2.1. Animals and Diets

A total of thirty-six nine-parity sows (Landrace × Yorkshire), with initial body weights (BW) of 233.56 ± 2.77 kg and backfat thickness (BF) <14 mm at day 0 of gestation were used in this study. Sows were artificially inseminated with pooled semen obtained from a littermate boar (housed on the research farm) on the day of estrus, and then 12 and 24 h later. After artificial insemination, sows were randomly assigned to one of two diets: the control (CON) diet was designed as per the recommendations of the National Research Council (NRC; 2012) for gestating sows, and contained 3.40 MCal digestible energy (DE) per kilogram, and 7.3% crude protein, together with 5% crude fiber; while the low-energy diet (LED) contained 3.00 MCal DE/kg, with the same crude protein and crude fiber as CON. Sows were fed 2.2 kg/day during early pregnancy (days 1–30 of gestation), 2.4 kg/day mid-pregnancy (days 31–90), and 2.8 kg/day late pregnancy (days 91 to parturition). After farrowing, sows were fed a lactation diet in accordance with NRC 2012, and were offered the diet three times per day (i.e., at 0800, 1200, and 1500 h), starting at 2.0 kg/day, then gradually increasing by 1 kg/day until the sixth day, after which the sows could feed *ad libitum*. Throughout the experiment, all animals had free access to water. The ambient temperature for sows was maintained at 20–25 °C. Heating light and pads were provided for suckling piglets, and the temperature was maintained at 26–32 °C, which was gradually decreased with increase in age. All sows were housed in individual feed stalls during gestation and, during lactation, were housed in farrowing pens.

At birth, six piglets (one piglet from each of six litters), each having approximately the same body weight (±10%) as the mean body weight of the litter, were selected from each group and slaughtered before suckling. At the day of weaning (day 28 post-birth), twelve healthy male piglets were selected from each of the two treatments. Six piglets per treatment were intraperitoneally injected with LPS solution to administer LPS at 50 µg/kg body weight, in order to make a model of immunological challenge, and another six piglets in each treatment were intraperitoneally injected with an equivalent amount of sterile saline. Feed was removed 4 h before the injection. The rectal temperature of each piglet was measured every 2 h. LPS (*Escherichia coli* L2880, Sigma-Aldrich, Los Angeles, CA, USA) was dissolved in sterile saline (9 g/L) to make the LPS solution (500 mg LPS per liter of saline). The dosage of the LPS injection and the time to sacrifice piglets were described previously [16].

### 2.2. Sample Collection

The pigs were anesthetized with an intravenous injection of pentobarbital sodium (50 mg/kg BW) before blood sample collection, and then slaughtered. From each group, six newborn piglets without eating colostrum (from six sows and close to the average body weight in each treatment group, three males and three females) were slaughtered, as well as 12 weaning piglets deprived of breast nursing from sows for 4 h before the LPS injection (12 males from 12 different sows, and close to the average body weight in each treatment group). On the day of birth and at 0 and 4 h after injection of LPS or normal saline, blood samples were collected from the anterior vena cava and placed into heparinized tubes, centrifuged (2550× *g*, 4 °C, 10 min) and stored at −20 °C until analysis. Immediately after collection of blood samples at 4 h, piglets were slaughtered by jugular puncture, and the entire intestine was removed as quickly as possible. The small intestine (SI) was quickly measured for weight and length. The middle portion (approximately 4 cm) of each segment of the small intestine (duodenum, jejunum, and ileum) was rapidly obtained and placed in liquid nitrogen, and then stored at −80 °C for subsequent analysis, as previously described [17]. In addition, about 1 cm each of integral duodenum, jejunum, and ileum tissue were cut out, fixed with 4% paraformaldehyde for intestinal slice production and morphology observation. The intestinal mucosa of the remaining duodenum, jejunum, and ileum segment was obtained carefully with a glass slide scraping, frozen in liquid nitrogen as soon as possible, and then stored at −80 °C for future analysis of intestinal enzyme activity. Colonic and caecal content samples were collected quickly, as previously described [18], and then stored at −80 °C for future analysis of short-chain fatty acids and intestinal microflora.

### 2.3. Intestinal Morphology Analysis

Intestinal tissue samples were fixed with 4% formaldehyde, dehydrated stepwise with ethanol (from low concentration to high concentration, up to 100%), and then embedded in transparent paraffin and sliced [17]. All tissue sections (5 μm) were stained with hematoxylin and eosin (HE) and mounted with gum. The slice images were captured at 100× magnification using an Olympus BX51 microscope equipped with a DP70 digital camera (Olympus, Tokyo, Japan). Crypt depth (μm) and villus length (μm) were measured with Image-Pro Plus 6.0 software (Media Cybernetics, Bethesda, MD, USA). At least eight pairs of villus and crypts were observed in each slice, and the average was the final value.

### 2.4. Analysis of Intestinal Enzyme Activity

Intestinal enzyme activity was measured as previously described [17]. After thawing, 0.2–0.5 g of mucosa samples were homogenized according to the weight of intestine mucosa (g): volume of physiological saline pre-cooled on ice (mL) = 1:9, centrifuged at 2500 g/min for 10 min, the supernatant was used to measure enzymatic activity. The enzyme activity was measured strictly in accordance with the respective instructions of the kit (Jiancheng Bioengineering Ltd., Nanjing, China).

### 2.5. RT-PCR Analysis

Gene expression levels were detected by using a reverse transcription polymerase chain reaction (RT-PCR) similar to the procedure previously described [19]. Briefly, total RNA was extracted from frozen ileum samples with TRIzol reagent (Invitrogen, Carlsbad, CA, USA), synthesis of cDNA from isolated total RNA was carried out with Prime-Script™ RT reagent kit (Takara, Dalian, China) according to the instructions of the manufacturer. Real-time PCR assays were performed on complementary DNA samples in 384-well optical plates on a 7900HT ABI Prism Sequence Detection System (Applied Biosystems, Foster City, CA, USA) using the SYBR green system (Takara). Primers for individual genes were designed using Primer Express 3.0 (Applied Biosystems, Foster City, CA, USA). The reaction mixture (10 μL) contained 5 μL of fresh SYBR^®^Premix Ex Taq II (Tli RNaseH Plus) and 0.2 μL ROX Reference Dye II (50×), 0.8 μL of the primers, 1 μL of RT products and 3 μL diethylpyrocarbonate-treated water. The PCR protocol was used as follows: 1 cycle (95 °C for 30 s), 40 cycles (95 °C for 5 s, 60 °C for 31 s) and 1 cycle (95 °C for 15 s, 60 °C for 1 min and 95 °C for 15 s). The relative mRNA abundance of the analyzed genes was calculated using the 2^−ΔΔCt^ method, as described previously by Mou et al. [19]. The most stable housekeeping genes (β-actin and GADPH) were chosen for normalization. Finally, the mRNA level of each target gene for the CON group was set to 1. The primer sequence is shown in Table 1.

### 2.6. Plasma TNF-α, IL-1β, IL-10, IgA and GLP-2 Analysis

Plasma TNF-α, IL-1β, IL-10, IgA and GLP-2 concentrations were measured by using ELISA kits suitable for pigs (Nanjing JianCheng Bioengineering Institute, Inc., Nanjing, China) according to the manufacturer’s protocol.

### 2.7. Plasma IgG and IgM Analysis

Routine analysis of blood samples in heparinized-plasma venipuncture vials were performed by automatic blood analyzer (Alfa Basic 16p, Boule Medical AB, Stockholm, Sweden).

### 2.8. Short Chain Fatty Acid (SCFA) Analysis

SCFAs, including acetate, propionate and butyrate in digesta and mucosa samples, were analyzed with a modification of the previous method [18]. Briefly, 2 g of digesta or mucosa samples were weighed into a 10-mL centrifuge tube and added to 5 mL of deionized water. After the tube was capped, the contents were vortex mixed for 30 s, left to stand for 30 min at 4 °C and then centrifuged (1000× *g*, 4 °C) for 10 min. The supernatant (1.2 mL) was removed by aspiration into another 5-mL centrifuge tube, added to 0.24 mL of 25% metaphosphate, vortex mixed for 30 s, and then left to stand for 30 min at 4 °C. Next, the contents were centrifuged (1000× *g*, 4 °C) for 10 min, and then 1.2 mL of the supernatant was removed by aspiration, added to 23.3 μL of 210 mmol/L cortonic acid, and vortex mixed for 30 s. Then, 0.3 mL of the mixed solution was removed and placed into another 2-mL tube, added to 0.9 mL Carbinol, and vortex mixed for 30 s for subsequent gas chromatography analysis. The samples were analyzed by CP-3800 gas chromatography (Varian, Inc., Artaud, CA, USA) equipped with a micro-injector (10 μL), a flame ionization detector and a capillary chromatographic column (CP-FFAP, 25 m × 0.32 mm × 0.3 μm). The injector temperature was 220 °C, the detector temperature was 250 °C, hydrogen flux was 40 mL/min, and air flux was 450 mL/min. The temperature program was as follows: 100 °C, hold 1 min, increase to 190 °C at 20 °C/min, increase to 190 °C, hold 3 min. Peaks were identified by comparing their retention times with individual reference standard fatty acids.

### 2.9. PCR Analysis of Bacteria

Quantitative RT-PCR of total bacteria was performed with SYBR^®^ Green PCR reagents (TaKaRa, Kyoto, Japan), whereas quantitative RT-PCR for Bifidobacterium, Lactobacillus, and Escherichia coli were performed with Taq primers and fluorescent oligonucleotide probes that had been commercially synthesized (Life Technologies Ltd., Beijing, China), as listed in Table 2 [20]. The PCR reaction of total bacteria was set up in a total volume of 15 μL and contained 7.5 μL of SYBR Premix Ex TaqII, 0.6 μL of each primer, 0.6 μL of genomic DNA, and 5.7 μL of sterile deionized water; whereas the PCR reactions for *Bifidobacterium*, *Lactobacillus*, and *Escherichia coli* were set up in a total volume of 10 μL, and contained 0.15 μL of probe, 0.5 μL of each primer, 0.5 μL of probe enhancer solution, 4 μL of Real Master Mix, 0.5 μL of genomic DNA, and 3.85 μL of sterile deionized water. Amplification was performed using a CFX 96 System (Bio-Rad Laboratories, Inc., Hercules, CA, USA). As for total bacteria, the amplification program consisted of one cycle of 95 °C for 30 s and then 40 cycles of 95 °C for 5 s, 60 °C for 34 s, and one cycle of 95 °C for 15 s, 60 °C for 1 min, 95 °C for 15 s. For *Bifidobacterium*, *Lactobacillus*, and *Escherichia coli*, the amplification program consisted of one cycle of 50 °C for 2 min, 95 °C for 10 min and then 40 cycles of 95 °C for 15 s and 55.6–59.5 °C for 1 min. Melting curve analysis and size determination of PCR fragment on agarose gels verified amplification of the target fragments. Standard curves were generated as previously described [20].

### 2.10. Statistical Analyses

SPSS 21.0 (IBM SPSS Company, Chicago, IL, USA) was used for all statistical analyses. Newborn piglets’ results were tested for variance using Levene’s test; a two-tailed *t*-test was used to compare the differences between the dietary groups. CON and the LED results after LPS challenge were performed by two-way ANOVA; data was tested for normality and homogeneity of variances (Shapiroe–Wilk and Levene tests, respectively) and, when necessary, data was normalized (arcsine, square root, or logarithm normalized) to achieve ANOVA assumptions. Additionally, Duncan’s multiple range tests were conducted to determine the difference among the treatments. All values are means with their standard errors (SE), and differences between treatments were considered significant when *p* < 0.05.

## 3. Results

### 3.1. BW and Small Intestinal Index

As shown in Table 3, the maternal low-energy diet significantly lowered the body weight of piglets at birth (*p* < 0.01). Maternal LED piglets had a higher ratio of SI length to BW than CON piglets (*p* < 0.01). We also found the maternal low-energy diet significantly reduced the ratio of SI weight to length in newborn piglets (*p* < 0.05). However, maternal diet did not affect the weight of the small intestine (SI), the length of the SI, or the ratio of SI weight to BW in the newborn piglets (*p* > 0.05).

In weaning piglets, maternal diet significantly lowered BW, SI weight, SI length, and SI weight/length (*p* < 0.001, *p* < 0.001, *p* < 0.05 and *p* < 0.001, respectively) (Table 4). SI weight/length of weaning piglets was also significantly affected by LPS challenge (Table 4). Compared with −LPS LED group and +LPS LED group, SI weight/BW was higher in -LPS CON group (*p* = 0.001).

### 3.2 Intestinal Morphology

**Figure 1:**
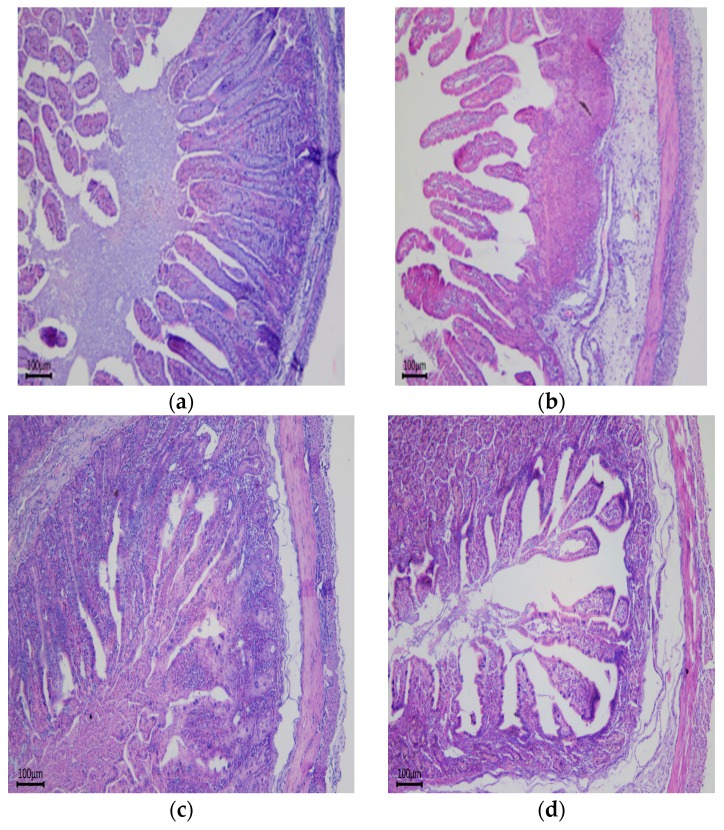
Jejunal histomorphology of newborn and weaning piglets. (**a,c**) indicate the control diet (CON); (**b**,**d**) indicate the low-energy diet (LED). (**a**,**b**) show the newborn piglets’ jejunal histomorphology; (**c**,**d**) show the weaning piglets’ jejunal histomorphology. Intestinal villi in (**b**,**d**) were shorter compared with the same period (**a**,**c**). Original magnification: 200×. (*n* = 6 for each group).

The villus height of jejunum and ileum, and the VCR (ratio of villus height to crypt depth) of the duodenum and ileum were lower in LED newborn piglets than in CON piglets (*p* < 0.05, Table 5 and Figure 1).

Maternal diet significantly affected duodenum villus height and crypt depth (CD), jejunum villus height and ileum VCR of the weaning piglets (*p* < 0.001, *p* < 0.05, *p* < 0.01 and *p* < 0.05, respectively), but not by LPS challenge, the duodenum villus height (*p* < 0.05) was also significantly influenced by Diet × LPS (Table 6 and Figure 1). Compared with the CON treatment, lower jejunum villus height and ileum VCR was observed for the +LPS LED treatment (*p* < 0.05). The duodenum crypt depth in −LPS LED piglets was lower than CON piglets (*p* < 0.05), whereas there was no difference in +LPS LED piglets.

### 3.3. Disaccharidase Activity

The activity of lactase in jejunum and sucrase in ileum markedly decreased (*p* < 0.05) in the LED group in newborn piglets (Table 7).

In weaning piglets, the activity of sucrase in jejunum was significantly decreased by maternal diet (*p* < 0.01), it is not affected by the LPS challenge and Diet × LPS (*p* > 0.05), the sucrase in the LED group was lower than in the CON group (*p* < 0.05, Table 8). The activity of maltase in jejunum tended to be affected by LPS challenge (*p* = 0.056), but was significantly influenced by maternal diet (*p* < 0.01, Table 8).

### 3.4. Intestinal Expression of Genes

**Figure 2:**
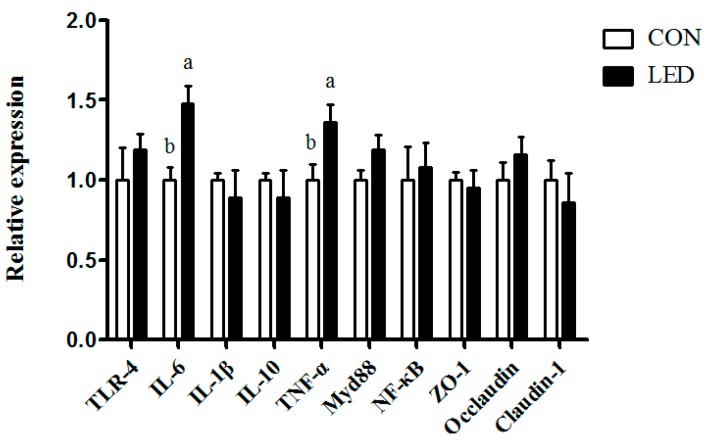
Effect of different energy levels during gestation on mRNA abundance of innate immune-related genes in ileum of newborn pigs. *TLR-4*, Toll-like receptor-4; *IL*, interleukin; *MyD88*, myeloid differentiation factor 88; *NF-κB*, nuclear factor kappa B; *TNF-α*, tumor necrosis factor α. Values are expressed as mean values with their standard errors; means with different superscript letters are significantly different (*p* < 0.05); the replicated number of each group is 6.

Maternal LED intake significantly up-regulated the mRNA relative expression of ileal *IL-6* and *TNF-α* in the newborn piglets (*p* < 0.01, Figure 2).

**Figure 3:**
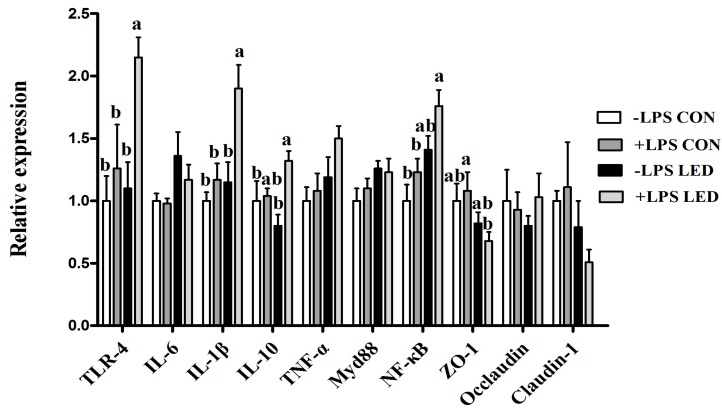
Effect of different energy level during gestation on immune related genes of weaned piglets challenged with Escherichia coli lipopolysaccharide. *TLR-4*, Toll-like receptor 4; *IL*, interleukin; *MyD88*, myeloid differentiation factor 88; *NF-κB*, nuclear factor kappa B; *TNF-α*, tumor necrosis factor α. Values are expressed as mean values with their standard errors; means with different superscript letters are significantly different (*p* < 0.05); the replicated number of each group is 6.

Maternal LED during gestation significantly affected the mRNA relative expression of ileal *IL-1β*, *NF-κB,* and *ZO-1* in weaning pigs. LPS challenge significantly affected mRNA abundance of *TLR-4*, *IL-1β*, *IL-10,* and *NF-κB* in ileum. The expression of ileal *IL-10* was also significantly affected by Diet × LPS interaction. The ileal *TLR-4* and *IL-1β* mRNA abundance in the +LPS LED group was higher than the remaining groups (*p* < 0.05). The ileal *IL-10* mRNA abundance in the +LPS LED group was higher than −LPS CON and −LPS LED groups (*p* < 0.05). Compared with +LPS CON group, the mRNA relative expression of ileal *ZO-1* was significantly decreased in the +LPS LED group (*p* < 0.05, Figure 3).

### 3.5. Plasma GLP-2 Concentration and GLP-2R Gene Expression in the Ileum

**Figure 4:**
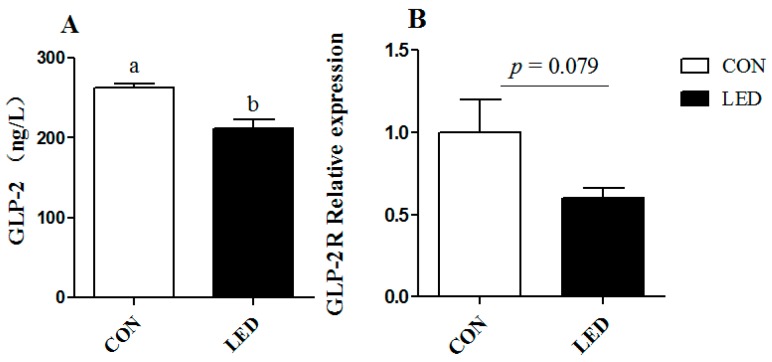
Effect of different energy level during gestation on plasma GLP-2 concentration (**A**) and *GLP-2R* mRNA expression (**B**) in the ileum of newborn pigs. Values are expressed as mean values with their standard errors; means with different superscript letters are significantly different (*p* < 0.05); the replicated number of each group is 6.

The plasma GLP-2 concentration was lower in LED newborn piglets than in CON piglets (*p* < 0.05, Figure 4A). The maternal LED intake group tended (*p* = 0.079) to decrease the mRNA relative expression of the ileal *GLP-2R* in the newborn piglets (Figure 4B).

**Figure 5:**
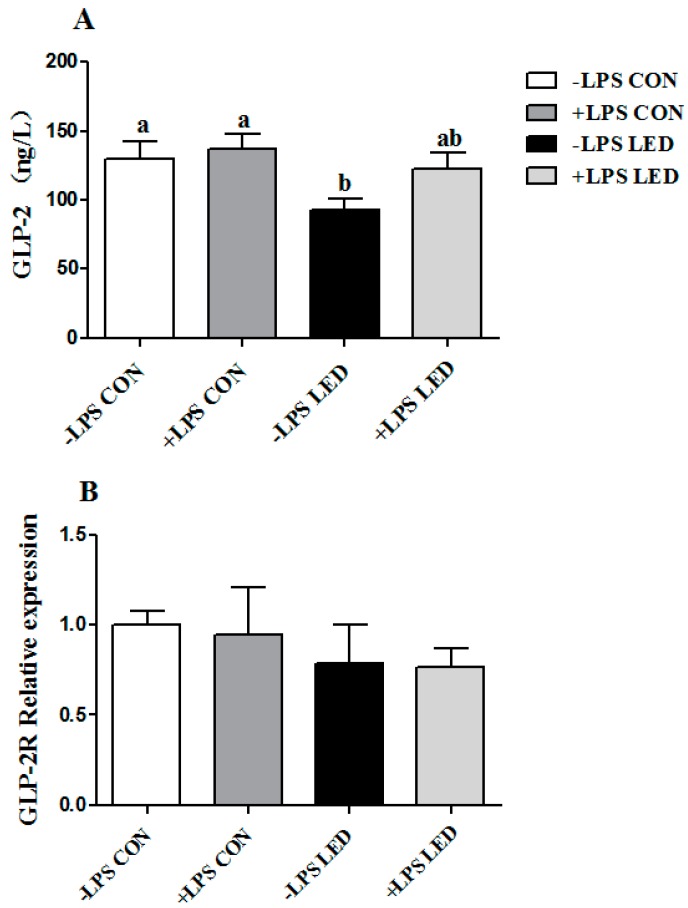
Effect of different energy levels during gestation on plasma GLP-2 concentration (**A**) and GLP-2R mRNA expression (**B**) in intestines of weaned piglets challenged with Escherichia coli lipopolysaccharide. Values are expressed as mean values with their standard errors; means with different superscript letters are significantly different (*p* < 0.05); the replicated number of each group is 6.

Plasma GLP-2 concentration was significantly affected by maternal diet in the weaning piglets (*p* < 0.01) (Figure 5A), whereas ileal *GLP-2R* mRNA abundance was not affected by maternal diet, LPS, or the interaction of DIET × LPS (Figure 5B). The plasma GLP-2 concentration in the –LPS LED group was lower than the CON groups (*p* < 0.05).

### 3.6. Changes of Body Temperature

LPS challenge resulted in increased (*p* < 0.001) rectal temperature at 4 h (Table 9).

### 3.7. Plasma Immunoglobulin Concentrations

**Figure 6:**
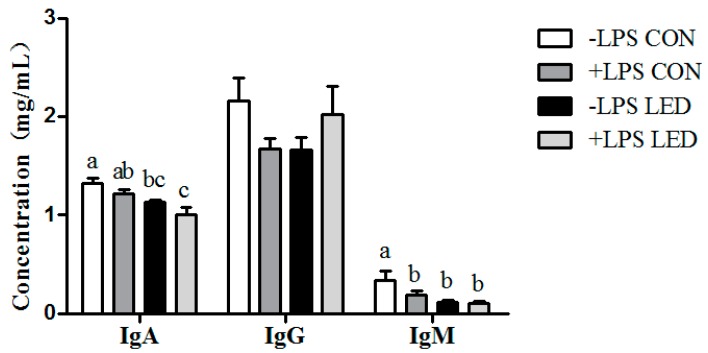
Effect of different energy levels during gestation on plasma immunoglobulin concentrations of weaned piglets challenged with Escherichia coli lipopolysaccharide. Values are expressed as mean values with their standard errors; means with different superscript letters are significantly different (*p* < 0.05); the replicated number of each group is 6.

Plasma IgA concentration was affected by maternal diet (*p* < 0.01) and LPS challenge (*p* < 0.05), while plasma IgM concentration was affected by maternal diet (*p* < 0.01) and Diet × LPS interaction (*p* < 0.01), and a tendency (*p* < 0.10) towards decrease was also observed following LPS challenge (Figure 6). Plasma IgA concentration was lower (*p* < 0.05) in the LED group compared with −LPS CON group. Compared with −LPS CON group, the plasma IgM concentration significantly decreased in the remaining groups at weaning (*p* < 0.05).

### 3.8. Plasma IL-1β, IL-10 and TNF-α Concentrations

**Figure 7:**
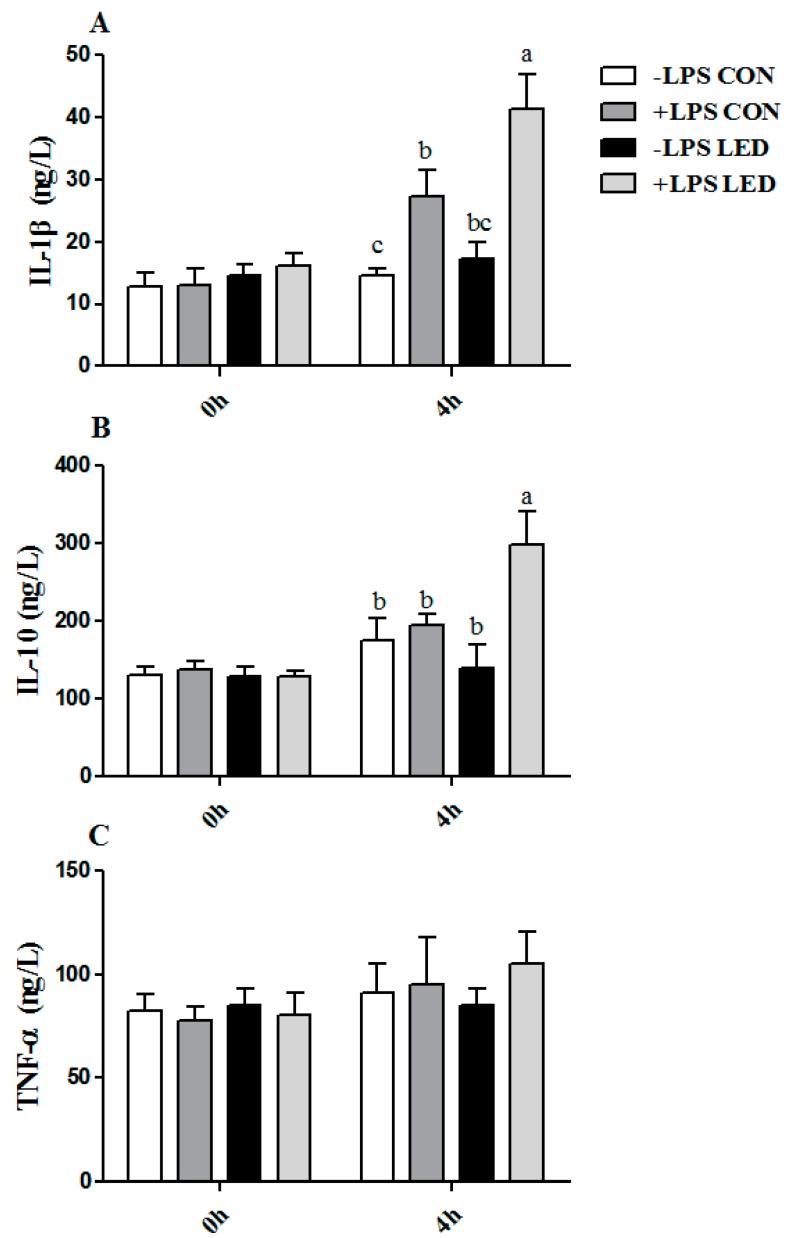
Effect of different energy levels during gestation on plasma IL-1β (**A**), IL-10 (**B**), and TNF-α (**C**) concentrations of weaned piglets challenged with Escherichia coli lipopolysaccharide. Values are expressed as mean values with their standard errors; means with different superscript letters are significantly different (*p* < 0.05); the replicated number of each group is 6.

Plasma IL-1β concentration was significantly affected by maternal diet (*p* < 0.05), LPS challenge (*p* < 0.001), and Diet × LPS interaction (*p* < 0.05) (Figure 7A), while plasma IL-10 concentration was significantly affected by LPS challenge (*p* < 0.01) and Diet × LPS interaction (*p* < 0.05) (Figure 7B). Plasma IL-1β and IL-10 concentrations were higher (*p* < 0.05) in the +LPS LED than in the remaining groups. In addition, plasma IL-1β concentration was higher (*p* < 0.05) in the +LPS CON group than in the −LPS CON group.

### 3.9. SCFA Concentrations

Acetate and propionic acid concentrations in caecal content were significantly decreased by LPS challenge (*p* < 0.05) (Table 10). Acetate concentration in caecal content was lower (*p* < 0.05) in the +LPS treatments than in the −LPS CON treatment, whereas propionic acid concentration in caecal content was lower (*p* < 0.05) in the +LPS LED group than in the −LPS CON group.

### 3.10. Bacteria Population in Colonic Content

Maternal diet and LPS challenge significantly increased population of *Escherichia coli* in colonic content (*p* < 0.05) (Table 11).

## 4. Discussion

In the embryonic period, the gastrointestinal system is one of the earliest systems of polarization, and the last few weeks before the birth of the fetus are a very important period for intestinal potential and digestive function maturity [21]. Some studies have shown that maternal nutrition affects the development and function of the gut of the offspring [5,6,16]. In this study, maternal LED intake during gestation significantly decreased piglet BW and SI indices, such as SI length and the SI weight-to-length ratio in newborn piglets. Piglets with lower birth weight may suffer from IUGR, which impairs growth, muscle accretion, duodenal mucosa morphology and carcass traits [22]. Previous studies have shown that IUGR piglets’ SI weight-to-length ratio is only 61–76% that of normal pigs [23,24], and that they have a lower SI mass [7] and SI length [25] at birth. Maternal pregnancy intake of nutrients, in addition to meeting their own needs, must also provide the fetus with nutrients to meet its growth and development [26]. Studies have shown that, compared with normal-weight piglets, IUGR reduced the growth rate of piglets one month after birth by about 15–30% [21]. The results of our study showed that maternal LED diets significantly decreased weaning weight, SI weight and length, SI unit length and SI weight to length ratio in weaned piglets. Studies in rats also showed a significant reduction in body weight, body length and intestinal length in low protein-to-energy rats compared to the control group, and decreased bowel weight and DNA in malnourished rats [27].

Integrity of the intestinal structure is required for the maintenance of intestinal nutrition [28]. Fetuses receive constant nutrients from mothers via the placenta, whereas pups must take up nutrients from food via the small intestine post-birth; therefore, small intestine development during the gestation period plays an important role. Previous studies have shown that low birth weight piglets have significantly decreased duodenal VCR, jejunum villus height and VCR compared with normal-weight piglets [29,30]. Some studies also showed that the small intestine surface area of IUGR piglets is smaller than that of normal pigs, mainly due to the lower average number of villi per unit area and low villus height. In our study, LED in sows during pregnancy significantly decreased the VCR of duodenum and ileum in newborn piglets, which was consistent with previous reports. Similarly, the results of this test found that sows fed a low-energy diet during pregnancy significantly decreased ileum VCR of weaning piglets. To our knowledge, the VCR represents the capacity of the small intestine for nutrient digestion and absorption [31]. The proliferation and differentiation of intestinal epithelial cells is accompanied by their migration along the crypt-villus axis [32]. Thus, a lower VCR may indicate less proliferation and differentiation of intestinal epithelial cells. It is logical to consider that maternal LED may suppress digestion and absorption function by inhibiting the proliferation and differentiation of intestinal epithelial cells in offspring. Consistently with the intestinal morphology results, we found jejunum mucosal lactase activity was significantly decreased in newborn piglets, which indicated digestion ability for lactose. Meanwhile, we also found jejunum mucosal sucrase and maltase activity were significantly decreased in weaning piglets. In terms of digestive capacity, intestinal enzymes are partially responsible for food processing and hydrolyzing macromolecule nutrients to small molecules for intestinal absorption [15]. During intrauterine life, glucose and amino acids are the main nutrients that provide energy for intrauterine growth; however, piglets utilize the abundant lactose in milk to provide most of their energy during neonatal life [33,34]. Based on this information, it may be concluded that intestinal digestion and absorption ability could be significantly decreased by a maternal low-energy diet during gestation.

Nutritional and other molecular events in fetal and neonatal life lay the foundation for future health, which includes effects on immune system function. The second aim of this study was to determine the potential effect of maternal LED on the immune responses of LPS-challenged piglets. The increased body temperature, plasma TNF-α, and IL-1β concentrations indicated the successful establishment of the immune model following LPS challenge. It has been demonstrated that the TLR-4–Myd88–NF-kB signal pathway is involved in inflammation [35]. Moreover, the gastrointestinal tract is the largest immune organ in the body and, as such, is the location for the majority of lymphocytes and immune effector cells with pattern recognition receptors [36], which sense luminal antigens and mediate the inflammatory response [37]. In the present study, ileal mRNA abundance of pro-inflammatory cytokines, including *TNF-α* and *IL-6*, were significantly up-regulated by maternal LED in newborn piglets, and plasma IgA and IgM were significantly reduced by the maternal dietary treatment in weaning piglets. Meanwhile, it was observed that LPS challenge resulted in increased plasma IL-1β concentration, which was consistent with gene expression results. Meanwhile, LPS challenge and low-energy diets increased *TLR-4* and *NF-kB* gene expression in weaning pigs’ ileum, which corresponded positively with inflammatory response. Previous studies also found maternal restraint stress during gestation in pigs enhanced the magnitude of the TNF-α and IL-6 responses to LPS in the offspring [38]. Therefore, maternal LED increased systemic inflammation, reduced the amount of immunoglobulin in offspring and up-regulated the expression of inflammation-related genes in offspring, resulting in increased sensitivity to LPS.

The intestinal epithelial barrier includes the presence of a single epithelial cell (EC) layer, tight junctions (TJs) between ECs, and the presence of associated immune cells [39]. ZO-1, Occludin and Claudin-1 are the most important tight junction proteins, and up-regulation of expression of these proteins means a reduction in intestinal inflammatory disease risk [40]. On the contrary, down-regulated expression of these proteins will lead to increased intestinal permeability and harmful bacteria in the intestine, whereupon LPS and other harmful substances will pass easily through the intestinal epithelium and into the submucosa, causing intestinal inflammation, damaging the health of the body [41]. In addition, earlier studies have reported that the production of excessive proinflammatory cytokines can activate the apoptotic pathway, induce apoptosis, and reduce the tight junction barrier of the intestinal epithelium [42]. In the present study, maternal LED significantly down-regulated the expression of the *ZO-1* gene in weaning piglets. At the same time, maternal LED significantly reduced the GLP-2 levels in newborn and weaning piglet plasma. GLP-2 increases the rate of crypt cell proliferation and villus elongation, and reduces apoptosis, leading to improved barrier function [43]. Meanwhile, endogenous GLP-2 production increased to improve the intestinal barrier function. SCFA (acetate, propionic acid and butyric acid) are the major end products of bacterial metabolism in the large intestine [44]. In the present study, LPS challenge resulted in the suppression of microbial fermentation, which was evidenced by the acetate and propionic acid concentrations in the caecal content, which were decreased by LPS challenge. A novel study in a mouse model demonstrated that increased production of acetate inhibits translocation of the E. coli toxin from the gut lumen to the blood, which improves intestinal defense mediated by epithelial cells, and thereby protects the host against lethal infection [45]. The decreased acetate content might, in part, account for the serious congestion in the intestine and destruction of the intestinal epithelial barrier. A previous study has shown that IUGR alters the GIT barrier and affects the gut immune response, as well as whole-body metabolism [25]. These results suggested sows fed low-energy diets during pregnancy may cause adverse intestinal epithelial barrier function, leading to increased intestinal permeability and greater susceptibility to inflammation.

## 5. Conclusions

In conclusion, our study demonstrates that decreasing energy intake during gestation suppressed the offspring’s intestinal digestion and absorption function and increased susceptibility of the weaning piglets to LPS challenge.

## Figures and Tables

**Table 1:** Oligonucleotide primers used for a relative-quantitative real-time PCR analysis.

Genes	Sequences (5′-3′)	Gene Bank No.
*TLR-4*	F:AGAAAATATGGCAGAGGTGAAAGC	GQ304754
	R:CTTCGTCCTGGCTGGAGTAGA	
*IL-1β*	F:TCTGCCCTGTACCCCAACTG	NM214055.1
	R:CCAGGAAGACGGGCTTTTG	
*IL-6*	F:GATGCTTCCAATCTGGGTTCA	M80258.1
	R:CACAAGACCGGTGGTGATTCT	
*IL-10*	F:GCCTTCGGCCCAGTGAA	NM_214041.1
	R:AGAGACCCGGTCAGCAACAA	
*TNF-α*	F:TCTATTTTGGGATCATTGCCC	NM_214022.1
	R:CCAGCCCCTCATTCTCTTTCT	
*MyD88*	F:GTGCCGTCGGATGGTAGTG	NM001099923
	R:TCTGGAAGTCACATTCCTTGCTT	
*NF-κB*	F:TGCTGGACCCAAGGACATG	AK348766.1
	R:CTCCCTTCTGCAACAACACGTA	
*ZO-1*	F: CCGCCTCCTGAGTTTGATAG	AJ318101
	R: CAGCTTTAGGCACTGTGCTG	
*Occludin*	F: TTCATTGCTGCATTGGTGAT	NM_0011636471
	R: ACCATCACACCCAGGATAGC	
*Claudin-1*	F: TCTTAGTTGCCACAGCATGG	NM_001244539
	R: CCAGTGAAGAGAGCCTGACC	
*GLP2R*	F: CTTGAGGAGACAACACGGAA	NM_001246266.1
	R: AGAGGAATGAGGCCAGCATA	
*β-actin*	F: GGCGCCCAGCACGAT	DQ845171.1
	R: CCGATCCACACGGAGTACTTG	
*GAPDH*	F: TCGGAGTGAACGGATTTGGC	NM_001206359.1
	R: TGCCGTGGGTGGAATCATAC	

*TLR-4*, Toll-like receptor-4; *IL*, interleukin; *MyD88*, myeloid differentiation factor 88; *NF-κB*, nuclear factor kappa B; *TNF-α*, tumor necrosis factor -α; *ZO-1,* Zonula occludens 1; *GLP-2R*, glucagon-like peptide-2 receptor; GAPDH, glyceraldehyde-3-phosphate dehydrogenase.

**Table 2:** Oligonucleotide primers and probes used for bacteriological analysis.

Primers/Probes		Sequence (5′-3′)	Tm (°C)	Product Size (bp)
*Escherichia coli*	Forward	CATGCCGCGTGTATGAAGAA	57	96
	Reverse	CGGGTAACGTCAATGAGCAAA		
	Probe	AGGTATTAACTTTACTCCCTTCCTC		
*Lactobacilli*	Forward	GAGGCAGCAGTAGGGAATCTTC	55.7	126
	Reverse	CAACAGTTACTCTGACACCCGTTCTTC		
	Probe	AAGAAGGGTTTCGGCTCGTAAAACTCTGTT		
*Bifidobacterium*	Forward	CGCGTCCGGTGTGAAAG	57	121
	Reverse	CTTCCCGATATCTACACATTCCA		
	Probe	ATTCCACCGTTACACCGGGAA		
Total bacteria	Forward	ACTCCTACGGGAGGCAGCAG	64.5	200
	Reverse	ATTACCGCGGCTGCTGG		

**Table 3:** Effect of different energy levels during gestation on intestinal index in newborn piglets.

Item	Control	Low Energy	*p*-Value
Birth weight, kg	1.44 ± 0.11	1.05 ± 0.35	0.007
SI weight, g	42.67 ± 6.40	31.10 ± 1.79	0.130
SI length, cm	290.17 ± 20.58	300.03 ± 15.76	0.711
SI weight/BW, g × kg^−1^ BW	29.16 ± 3.37	29.72 ± 2.14	0.892
SI length/BW, cm × kg^−1^ BW	205.86 ± 16.19	285.66 ± 14.78	0.005
SI weight/length, mg/cm	141.34 ± 13.02	104.34 ± 5.78	0.027

SI, small intestine; BW, body weight. Mean values with their standard errors; *n* = 6 for each group.

**Table 4:** Effect of different energy levels during gestation on intestinal index of weaned piglets challenged with Escherichia coli lipopolysaccharide.

Item	Control		Low Energy		*p*-Value		
	−LPS	+LPS	−LPS	+LPS	Diet	LPS	Diet × LPS
Body weight, kg	7.52 ± 0.10 ^a^	7.40 ± 0.09 ^a^	5.84 ± 0.20 ^b^	5.55 ± 0.08 ^b^	<0.001	0.133	0.497
SI weight, g	353.79 ± 16.97 ^a^	313.37 ± 23.65 ^a^	200.22 ± 10.26 ^b^	193.67 ± 13.44 ^b^	<0.001	0.164	0.367
SI length, cm	805.75 ± 15.75 ^a^	783.50 ± 20.31 ^ab^	642.60 ± 11.64 ^b^	702.00 ± 10.86 ^ab^	0.017	0.687	0.380
SI weight/BW, g × kg^−1^ BW	47.53 ± 2.39 ^a^	41.86 ± 4.04 ^ab^	34.24 ± 1.15 ^b^	34.90 ± 2.37 ^b^	0.001	0.341	0.234
SI length/BW, cm × kg^−1^ BW	108.16 ± 1.79	104.58 ± 8.92	110.12 ± 3.78	126.52 ± 10.70	0.137	0.412	0.209
SI weight/length, mg/cm	439.42 ± 20.14 ^a^	399.87 ± 10.79 ^a^	312.98 ± 17.21 ^b^	278.69 ± 14.48 ^b^	<0.001	0.039	0.873

SI, small intestine; BW, body weight. −LPS, piglets not challenged with LPS; +LPS, piglets challenged with LPS. Mean values with their standard errors. Within a row, means with different superscript letters are significantly different (*p* < 0.05); *n* = 6 for each group.

**Table 5:** Effect of different energy level during gestation on intestinal morphology in newborn pigs.

Item	Control	Low energy	*p*-Value
Duodenum			
Villus height (μm)	566.37 ± 37.57	529.08 ± 23.18	0.418
Crypt depth (μm)	135.81 ± 9.04	150.67 ± 7.84	0.243
VCR	4.21 ± 0.25	3.53 ± 0.16	0.048
Jejunum			
Villus height (μm)	635.46 ± 37.54	492.27 ± 45.22	0.041
Crypt depth (μm)	119.35 ± 2.24	107.08 ± 2.50	0.325
VCR	5.50 ± 0.35	4.61 ± 0.45	0.157
Ileum			
Villus height (μm)	782.46 ± 46.08	426.58 ± 51.45	0.001
Crypt depth (μm)	113.89 ± 7.80	112.90 ± 14.07	0.952
VCR	6.90 ± 0.14	3.80 ± 0.15	0.000

VCR, ratio of villus height to crypt depth. Mean values with their standard errors. *n* = 6 for each group.

**Table 6:** Effect of different energy levels during gestation on intestinal morphology of weaning piglets challenged with Escherichia coli lipopolysaccharide.

Item	Control		Low Energy		*p*-Value		
	−LPS	+LPS	−LPS	+LPS	Diet	LPS	Diet × LPS
Duodenum							
Villus height (μm)	491.46 ± 26.86 ^a^	423.46 ± 12.61 ^b^	335.90 ± 22.63 ^c^	374.25 ± 15.86 ^bc^	<0.001	0.454	0.015
Crypt depth (μm)	201.72 ± 10.82 ^a^	194.73 ± 7.25 ^a^	169.07 ± 7.28 ^b^	191.22 ± 4.72 ^ab^	0.031	0.332	0.074
VCR	2.47 ± 0.26	2.38 ± 0.40	1.99 ± 0.12	2.01 ± 0.25	0.173	0.909	0.845
Jejunum							
Villus height (μm)	322.18 ± 13.96 ^a^	314.34 ± 13.69 ^a^	289.17 ± 16.72 ^ab^	260.96 ± 12.31 ^b^	0.008	0.224	0.485
Crypt depth (μm)	133.11 ± 9.43	145.35 ± 8.47	148.60 ± 6.90	140.59 ± 7.83	0.534	0.805	0.248
VCR	2.44 ± 0.26	2.20 ± 0.20	1.98 ± 0.19	1.92 ± 0.28	0.157	0.555	0.707
Ileum							
Villus height (μm)	272.60 ± 9.15	260.27 ± 12.58	246.79 ± 18.65	225.71 ± 18.38	0.066	0.287	0.776
Crypt depth (μm)	121.55 ± 9.13	114.92 ± 11.79	127.65 ± 2.89	144.82 ± 10.63	0.128	0.642	0.302
VCR	2.26 ± 0.10 ^a^	2.36 ± 0.22 ^a^	1.95 ± 0.24 ^ab^	1.62 ± 0.17 ^b^	0.019	0.569	0.290

VCR, ratio of villus height to crypt depth. −LPS, piglets not challenged with LPS; +LPS, piglets challenged with LPS. Mean values with their standard errors. Within a row, means with different superscript letters are significantly different (*p* < 0.05); *n* = 6 for each group.

**Table 7:** Effect of different energy levels during gestation on intestinal enzyme activity in newborn pigs.

Item	Control	Low Energy	*p*-Value
Jejunum			
Lactase (U/mg protein)	161.28 ± 20.13	83.95 ± 14.58	0.010
Sucrase (U/mg protein)	3.03 ± 0.43	1.92 ± 0.42	0.167
Maltase (U/mg protein)	15.59 ± 3.38	14.14 ± 2.66	0.550
Ileum			
Lactase (U/mg protein)	23.92 ± 3.71	15.48 ± 2.08	0.717
Sucrase (U/mg protein)	1.43 ± 0.23	0.71 ± 0.13	0.020
Maltase (U/mg protein)	6.55 ± 1.40	9.19 ± 1.22	0.401

Mean values with their standard errors. *N* = 6 for each group.

**Table 8:** Effect of different energy levels during gestation on intestinal enzyme activity of weaning piglets challenged with Escherichia coli lipopolysaccharide.

Item	Control		Low Energy		*p*-Value		
	−LPS	+LPS	−LPS	+LPS	Diet	LPS	Diet × LPS
Jejunum							
Lactase (U/mg protein)	80.26 ± 13.24	77.45 ± 12.76	97.26 ± 9.20	83.08 ± 7.91	0.340	0.469	0.623
Sucrase (U/mg protein)	90.28 ± 11.95 ^a^	86.78 ± 7.58 ^a^	56.91 ± 6.97 ^b^	54.13 ± 9.13 ^b^	0.002	0.729	0.969
Maltase (U/mg protein)	179.39 ± 10.27 ^a^	137.82 ± 12.99 ^b^	126.08 ± 5.21 ^b^	113.77 ± 16.43 ^b^	0.009	0.056	0.282
Ileum							
Lactase (U/mg protein)	15.91 ± 1.65	13.15 ± 1.27	11.24 ± 2.25	12.16 ± 1.48	0.110	0.585	0.286
Sucrase (U/mg protein)	45.21 ± 10.40	44.82 ± 9.16	38.44 ± 4.52	43.16 ± 6.00	0.597	0.785	0.748
Maltase (U/mg protein)	87.85 ± 6.87	82.28 ± 11.60	67.51 ± 12.65	62.41 ± 12.63	0.063	0.604	0.982

−LPS, piglets not challenged with LPS; +LPS, piglets challenged with LPS. Mean values with their standard errors. Within a row, means with different superscript letters are significantly different (*p* < 0.05); *n* = 6 for each group.

**Table 9:** Effect of different energy levels during gestation on temperature of weaned piglets challenged with Escherichia coli lipopolysaccharide.

Time	Control		Low Energy		*p*-Value		
	−LPS	+LPS	−LPS	+LPS	Diet	LPS	Diet × LPS
0 h (°C)	39.75 ± 0.08	39.70 ± 0.06	39.63 ± 0.13	39.73 ± 0.10	0.665	0.795	0.439
4 h (°C)	39.67 ± 0.06 ^b^	41.23 ± 0.27 ^a^	39.62 ± 0.07 ^b^	41.23 ± 0.15 ^a^	0.817	<0.001	0.817

−LPS, piglets not challenged with LPS; +LPS, piglets challenged with LPS. Mean values with their standard errors. Within a row, means with different superscript letters are significantly different (*p* < 0.05); *n* = 6 for each group.

**Table 10:** Effect of different energy level during gestation on SCFA of weaned piglets challenged with Escherichia coli lipopolysaccharide.

Item	Control		Low Energy		*p*-Value		
	−LPS	+LPS	−LPS	+LPS	Diet	LPS	Diet × LPS
Caecal content, μmol/g							
Acetate	35.63 ± 2.76 ^a^	21.53 ± 3.74 ^b^	29.15 ± 4.28 ^ab^	21.09 ± 5.28 ^b^	0.432	0.021	0.491
Propionic acid	12.07 ± 0.94 ^a^	8.27 ± 1.54 ^ab^	10.26 ± 1.46 ^ab^	7.03 ± 1.81 ^b^	0.251	0.025	0.702
Butyric acid	4.58 ± 0.76	3.27 ± 0.50	4.60 ± 0.49	4.75 ± 1.51	0.436	0.546	0.449
Colonic content, μmol/g							
Acetate	14.80 ± 3.71	11.00 ± 2.23	12.63 ± 1.88	10.11 ± 2.37	0.566	0.243	0.808
Propionic acid	5.47 ± 1.36	4.36 ± 1.31	4.71 ± 1.02	3.04 ± 0.71	0.372	0.239	0.811
Butyric acid	2.58 ± 0.45	1.82 ± 0.59	1.86 ± 0.31	1.39 ± 0.46	0.262	0.227	0.770

−LPS, piglets not challenged with LPS; +LPS, piglets challenged with LPS. Mean values with their standard errors. Within a row, means with different superscript letters are significantly different (*p* < 0.05); *n* = 6 for each group.

**Table 11:** Effect of different energy level during gestation on intestinal bacteria of weaned piglets challenged with Escherichia coli lipopolysaccharide.

Item	Control		Low Energy		*p*-Value		
	−LPS	+LPS	−LPS	+LPS	Diet	LPS	Diet × LPS
*Escherichia coli*	6.18 ± 0.16 ^b^	6.37 ± 0.15 ^b^	6.31 ± 0.19 ^b^	6.94 ± 0.11 ^a^	0.040	0.020	0.178
*Lactobaciilus*	8.16 ± 0.14	7.98 ± 0.27	8.00 ± 0.31	7.77 ± 0.17	0.632	0.621	0.963
*Bifidobacterium*	7.42 ± 0.39	7.21 ± 0.21	7.10 ± 0.64	6.91 ± 0.86	0.646	0.759	0.996
Total bacteria	10.52 ± 0.13	10.60 ± 0.18	10.22 ± 0.29	10.43 ± 0.19	0.267	0.498	0.754

−LPS, piglets not challenged with LPS; +LPS, piglets challenged with LPS. Mean values with their standard errors. Within a row, means with different superscript letters are significantly different (*p* < 0.05); *n* = 6 for each group.
